# Phenotypic and genotypic drug sensitivity profiles of *Mycobacterium tuberculosis* infection and associated factors in northeastern Ethiopia

**DOI:** 10.1186/s12879-021-05961-8

**Published:** 2021-03-12

**Authors:** Fikru Gashaw, Berhanu Erko, Yalemtsehay Mekonnen, Bazezew Yenew, Misikir Amare, Balako Gumi, Gobena Ameni

**Affiliations:** 1 Aklilu Lemma Institute of Pathobiology, Addis Ababa University, P.O. Box 1176, Addis Ababa, Ethiopia; 2 Department of Microbial, Cellular and Molecular Biology, College of Natural Sciences, Addis Ababa University, P.O. Box 1176, Addis Ababa, Ethiopia; 3 Department of Biology, College of Natural and Computational Sciences, Kotebe Metropolitan University, P.O. Box 31248, Addis Ababa, Ethiopia; 4 Ethiopian Public Health Institute, P.O. Box 1242, Addis Ababa, Ethiopia; 5 Department of Veterinary Medicine, College of Food and Agriculture, United Arab Emirates University, Al Ain, P.O. Box 15551 Abu Dhabi United Arab Emirates

**Keywords:** *Mycobacterium tuberculosis*, Drug resistance, BACTEC MGIT 960, MTBDR*plus* assay, Northeastern Ethiopia

## Abstract

**Background:**

*Tuberculosis is* a devastating and a deadly *disease* despite the novel advances in its diagnostic tools and drug therapy. Drug resistant *Mycobacterium* contributes a great share to tuberculosis mortality. Status of drug resistance and patients’ awareness toward the disease is unknown in northeastern Ethiopia. Thus, the aim of this study was to determine the phenotypic and genotypic drug sensitivity patterns and associated factors in Oromia Special Zone and Dessie Town, northeastern Ethiopia.

**Methods:**

In a cross-sectional study, 384 smear positive tuberculosis cases were recruited and Löwenstein-Jensen culture was done. The performance of GenoTypic MTBDR*plus* assay using the conventional BACTEC MGIT 960 as a “gold standard” was determined. Drug resistant strains were identified using spoligotyping. Pearson Chi-square test was used to determine the association of drug sensitivity test and tuberculosis type, lineages, dominant strains and clustering of the isolates.

**Results:**

The 384 smear positive *Mycobacterium* samples were cultured on LJ media of which 29.2% (112/384) as culture positive. A fair agreement was found between MTBDR*plus* assay and the conventional MGIT test in detecting the *Mycobacterium tuberculosis* with sensitivity, specificity, positive and negative predictive value of 94.2, 30.2, 68.4 and 76.5%, respectively. Among LJ culture positive samples 95 of them gave valid result for MTBDR*plus* assay and 16.8% (16/95) as drug resistant. Similarly, MGIT subculture was made for the 112 isolates and 69 of them gave positive result with 15.9% (11/69) as drug resistant. Cohen’s kappa value showed almost a perfect agreement between the two testing methods in detecting rifampicin (sensitivity 100% and specificity 98.3%) and multi-drug resistance (sensitivity 83.3% and specificity 100%). Spoligotyping identified 76.5% (13/17) of the drug resistant isolates as Euro-American and family 33 as the predominant family. Significant association was observed between drug resistant isolates and the dominant strains (χ2: 34.861; *p* = 0.040) of the *Mycobacterium*.

**Conclusion:**

Higher magnitude of drug resistance was found in the study area. The GenoTypic MDRTB*plus* assay had an acceptable drug sensitivity testing performance.

## Background

Ethiopia is one of the 30 top global tuberculosis (TB) burden countries. Antimicrobial susceptibility testing is vital in prescribing an effective drug regime for TB patient, especially in areas where drug resistance incidence is high like Ethiopia. Although the global mortality rate of TB is reducing by 3% annually, the threat of its drug resistance is increasing [1]. The cumulative effects of treatment interruption like lack of awareness about the nature of bacteria, shortage and lack of WHOs recommended diagnostic tools, and prolonged drug consumption period for treatment increases the risk. Unless the new drug regimens are used, multidrug resistant (MDR) TB treatment could take more time causing longer absenteeism from work or even loss of employment, social isolation, and long-term socioeconomic and psychological effects [2].

In 2018, an estimated 3.4% of the global TB cases were new drug resistant (MDR/RR) and 18% were among previously treated cases. In Ethiopia, the estimated incidence of MDR/RR is 0.71 and 16% among new and previously treated cases, respectively [3]. In addition, the estimated cases of MDR-RR had a magnitude of 484,000 which was about 10% downward from the best estimate published by WHO in its 2018 global TB report. Of these estimated cases, about 44.2% (214,000) deaths were due to MDR/RR-TB which was also a downward revision to the best estimates. In fact, the global notified cases rather than estimates of MDR/RR-TB were 186,772 up from 160,772 cases in 2017, and 156,071 cases were enrolled in treatment which was also up from 139,114 in 2017. The number of people enrolled in treatment in the year was equivalent to only 32% of the estimated incidence of the 484,000 cases [3, 4].

The Genotype MTBDR*plus* assay diagnostic tools were used and correctly identified Rifampicin (RIF), Isoniazid (INH) and MDR-TB resistant isolates with best efficacy of sensitivity and specificity as shown in a study done at Cameroon [5]. The “End TB Strategy” calls early diagnosis of TB and prompt treatment for all persons of all ages with any form of drug susceptible or drug resistant TB. WHO further defines a universal access to those drug sensitivity testing technologies of both LPAs and *Mycobacterium* growth indicator tubes (MGIT). For proper treatment of the drug resistant tuberculosis on time a lesser time taking testing methods (GeneXpert, LPAs and MGITs) are recommended than the gold standard LJ culture method [6].

Spoligotyping is a widely used DNA fingerprinting methods that allows simultaneous detection and molecular typing of *M. tuberculosis* complex. It has a meticulous value in population based studies with low cost to define the phylogeographic specificity of the circulating clades/families of tubercle bacilli. It is the DR region used in individual *M. tuberculosis* strains and in different members of the *M. tuberculosis* complex to identify and align the spoligotype patterns. This imply that spoligotyping is recommended as the best preliminary screening test for *M. tb* isolates that circulates in the societies [7].

In Ethiopia, although great expansion of health institutes are built and primary attention is given to TB control and management, drug sensitivity testing is limited. Thus, the aim of this study was to assess the performance of both phenotypic and genotypic drug sensitivity test, status of drug resistance and the circulating resistant strains in Oromia Special Zone and Dessie Town administration in northeastern Ethiopia.

## Methods

### The study area and sample collection sites

The study was conducted in Oromia Special Zone (OSZ) and South Wollo Zone (SWZ) of the Amhara Regional State, northeastern Ethiopia. Kemise and Bati Town health centers were the data collection sites from OSZ where as Dessie Referral Hospital (DRH), Bikat Higher Diagnostic Laboratories (BHDL), Dessie Health Center (DHC) and Boru Meda Hospital (BMH) were the sample collection sites from South Wollo. Tuberculosis cases confirmed by the health personnel who fulfilled the inclusion criteria were included in the study. The samples (sputum and Fine needle aspirates (FNAs)) were collected on the spot from consenting participants 18 years and older of all TB cases as of April 2015 to January 2017. Those with severe TB who were unable to provide their sputum specimens were excluded from the study.

### Study design and laboratory processing

An institution-based cross-sectional study design was used among all forms of TB cases. Dry, translucent, leak-proof 50 ml capacities of falcon tubes were used to collect a minimum of 3-5 ml sputum sample and labeled using indelible labeling markers. The samples were collected from all those smear-positive TB confirmed participants of both previously untreated (new) and previously treated (retreatment) cases.

For bacteriological examination, direct microscopic examination was used after Ziehl-Neelsen staining technique performance at sample collection sites. A portion of Positive samples were kept at a range of − 10 to − 20 °C in the refrigerator of the health institutes. The FNAs from patients suspected for extra-pulmonary tuberculosis (EPTB) was collected using a 21-gauge needle attached to a 10 ml syringe with maximum care and safety by an experienced pathologist [8]. Afterwards, the Ziehl-Neelsen smear technique was performed by the same pathologist to check its positivity. A suction of about 1 ml samples was collected from positive patients for this research purpose and preserved in sterile and tightly closed nunc tubes with phosphate buffer saline of equal amount at pH 7.2 and kept in the same refrigerator [9]. Finally, both sputum and FNA samples were transported using a cold chain of 4 °C to TB laboratory of Aklilu Lemma Institute of Pathobiology (ALIPB) in Addis Ababa where the samples were kept in a deep freezer of – 80 °C until culturing was done [10].

A 37.2 g stock of selective Lowenstein-Jensen (LJ) media was suspended in 600 ml distilled water supplemented with 12 ml of glycerol. The glycerol was replaced by 10 g of sodium pyruvate for the second culturing media. Then frequent agitation was made for a minute by boiling. The final solution was cooled and sterilized in autoclave at 121 °C for 15 min. For the preparation of homogenized whole eggs, fresh hen eggs were washed with running water and sterilized by 70% ethanol before breaking to mix by shaking under aseptic condition. One liter of this homogenized egg was added to the prepared autoclaved media solution, shacked and sieved with sterile sieve. The completely prepared medium of 6-8 ml volume was dispensed into screw-capped culture tubes and their lids were securely fastened. Finally, the tubes were slanted at 15–20^0^ angles in inspissator and heated at 85 °C to solidify/coagulate for 45 min. The prepared media was left for 48 h before culturing the bacteria to test their contamination and those poor quality media were identified and discarded. Finally, the *Mycobacterium* culture was performed [11].

All the inoculated LJ slants in culture tubes were incubated aerobically at 37 °C in a slanted position and contamination was checked daily for the first week. The inoculated media was positioned in an upright position starting from the second week and colony formation of the bacteria was observed every week for eight consecutive weeks. The grown *M. tb* was heat killed and freezed whereas the weakly grown colonies were sub-cultured. Both the heat killed and freezed isolates were kept in a deep freezer of -80 °C until drug sensitivity test was done using both phenotypically and genotypically.

### The molecular GeneXpert and line probe assay

A total of 95 sputum samples were subjected to the Xpert test and the test was performed according to the manufacturer’s instruction in DRH. The automated readout reports the presence of *Mycobacterium* with a detection of rifampicin (RIF) resistance and recorded [12]. The GenoType MTBDR*plus* version 2 LPA was used for identification of *M. tuberculosis* complex and its resistance to RIF, isoniazid (INH) or both using the heat killed DNA extracts. The assay was based on the DNA-STRIP technology and the whole procedure involves a multiplex PCR amplification with biotinylated primers, and a reverse hybridization. This assay detected for the absence and/or presence of wild type (WT) and/or mutant (MUT) DNA sequences with in specific region of three genes: the rpoB gene (coding for the β-subunit of the RNA polymeraze), for the identification of RIF resistance; the katG gene (coding for the catalase peroxidase), for high level INH resistance; and the promoter region of the inhA gene (coding for the NADH enoylACP reductase), for low-level INH resistance. The procedure of the test was performed based on the manufacturer’s instructions. Missing of bands in *rpoB* probes showed resistance to RIF whereas missing of *katG* and/or *inhA* indicates INH resistance. On the other hand, the mutation probes were also considered as resistant when the bands are as strong as or stronger than the existing AC bands. Absence of the wild type (WT) band is usually accompanied by the presence of mutant (MUT), which indicates resistance. In rare cases, missing of WT band(s) without a corresponding MUT band might be observed which was considered as due to “uncommon mutations” in the probe region. Presence of both WT and MUT bands in the same stripe might be an indication for the presence of hetero-resistance or mixed infection [13].

### Phenotypic BD BACTEC™ MGIT™ 960 system (SIRE test)

Sub-culturing was performed for 112 LJ-culture positive freezed isolates using BACTEC MGIT 960 instrument (Becton Dickinson, Baltimore, MD, USA) for a maximum of 42 days from initial incubation date.. The drug sensitivity test to streptomycin (S), isoniazid (I), rifampicin (R) and ethambutol (E) was done for all of the successfully recovered sub-culture positive isolates following the protocols [14]. To control tubes, 0.5 ml of Growth Control working solution was added and labeled as drug free *Mycobacterium* growth indicator tubes (MGIT), and the other drugs containing tubes were labeled S, I, R and E. The result was interpreted as the growth unit (GU) of the growth control (GC) reached at a minimum value of 400. At the time the GU value of the GC was 400 or more and if the GU value of the drug tube was less than 100, the test result was reported as “susceptible,” while if the GU value of the drug tube was 100 or more the result was interpreted as “resistant”. The GU values of both the DST sets were retrieved and recorded. In case the GU value of the control did not reach 400 within 21 days, the instrument indicated an X200 error, indicating insufficient growth. On the other hand, if the GU reached 400 earlier than day 4, the instrument gave an X400 error, indicating contamination or over inoculation and in such cases the test was repeated [15]. In all cases of the study, the methods were carried out in accordance with relevant guidelines and regulations.

### Data analysis

The recorded data was checked for completeness and consistency, and then entered into Microsoft Excel 2007 spreadsheets. The data was then exported to IBM SPSS Statistics for Windows, Version 25.0. (Armonk, NY: IBM Corp., USA) program for analysis. Descriptive statistics was used to determine frequency and percentage. In spoligotyping, the reference data base (SpolDB4) available online through https://www.pasteur-guadeloupe.fr:8081/SITVITDemo/ was used to assign the shared international spoligotype numbers (SIT) to known profiles for the drug resistant isolates and if not available the new patterns were considered as ‘orphan’ types. An online tool Run TB-Lineage with a website of http://tbinsight.cs.rpi.edu/run_tb_lineage.html was also used to identify family/clade, lineages and sublineages of the isolates. Those identical spoligotypes patterns with identical DNA genotypes were considered as a cluster and these clustered strains were identified as an indicator for the recent transmission.

Sensitivity, specificity, positive and negative predictive values were calculated to compare the performances between drug sensitivity tests. Agreement between the tests was assessed by Cohen’s Kappa statistics. The Kappa value was interpreted with values < 0 as indicating no agreement and 0–0.20 as slight, 0.21–0.40 as fair, 0.41–0.60 as moderate, 0.61–0.80 as substantial, and 0.81–1 as almost perfect agreement [16]. The Pearson Chi-square (χ2) was used to determine the association of drug sensitivity test and TB type, lineages, dominant strains and clustering of the isolates. The results were considered as statistically significant when the *p*-value was less than 5%.

## Results

### Socio-demographic characteristics and tuberculosis infections

A total of 384 TB cases took part in the study with majority of the participants being males (55.5%). Ages of the study participants ranged from 18 to 75 years and the median age was 30 (IQR = 15). The prevalence of TB cases was highest (67.0%) in the 18–37 years age group. There was no significant difference among males (mean = 34.5, SD = 12.3) and females (mean = 32.7, SD = 11.4).

### GeneXpert MTB/RIF, GenoType MTBDR*plus* assay and BACTEC MGIT 960 test result

**Fig. 1:**
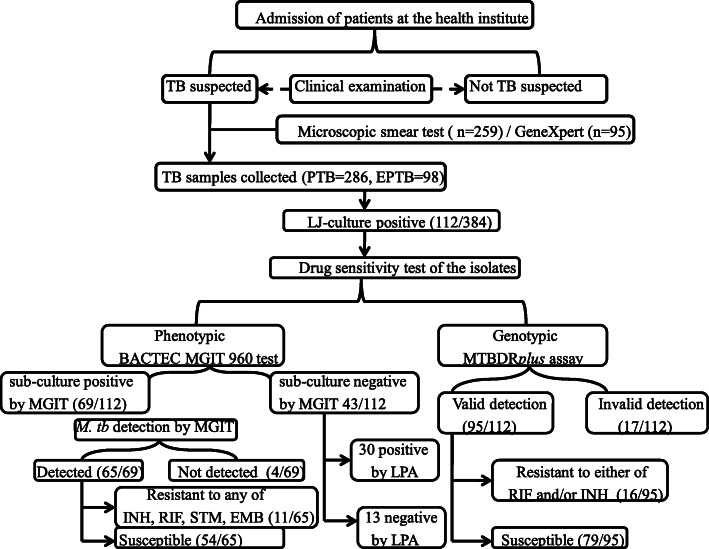
A flowchart outlining sample collection and the number of resistant isolates using both phenotypic and genotypic drug sensitivity test

The overall LJ-culture positive sample was 29.2% (112/384). Among the collected samples, 95 were identified by rapid molecular diagnostic GeneXpert at DRH while the rest were detected by smear microscopic test (Fig. 1). Of the Xpert MTB/RIF tested samples, two were identified as RIF resistant. Twenty two of the 95 GeneXpert samples were LJ-culture positive and used for further drug sensitivity test using the GenoType MTBDR*plus* assay and the conventional BACTEC MGIT 960 System. Both methods confirmed the two RIF resistant samples by Xpert as drug resistant and in addition they also detect one more sample as multidrug resistant.

The sub-culture test by MGIT gave 61.6% (69/112) as positive and 64 of these sub-culture positive samples as from new TB cases and the rest five as from retreatment patients. Sixty five samples were correctly identified as *M. tb* and 30 of the MGIT sub-culture negative samples were detected as *M. tb* positive by the GenoType MTBDR*plus* assay. The remaining 13 of the 112 LJ-culture positive samples were negative to MGIT sub-culture and also not detected as *M. tb* by LPA. Of the 112 isolates tested by line probe assay 15.2% (17/112) have no TUB band. The translated sensitivity, specificity, positive predictive value and negative predictive value were 94.2, 30.2, 68.4 and 76.5%, respectively. There was a fair agreement (Kappa = 0.276; *P* < 0.001) between the two methods (MGIT and LPA) in detecting the *Mycobacterium* (Table 1) when MGIT was used as the reference standard in the analysis [17].

**Table 1:** *M. tuberculosis* detection rate by line probe assay and *Mycobacterium* growth indicator tube using the 112 LJ-culture positive samples

LPA detection	MGIT detection		Total (=112)
	Detected (=69)	Not detected (=43)	
Detected	65	30	95 (84.8%)
Not detected	4	13	17 (15.2%)

Sensitivity test done by LPA detected 16.8% (16/95) as drug resistant of which 18.8% (3/16) were from retreatment cases. The four first line anti-TB drugs (STM, INH, RIF and ETM) by conventional BACTEC MGIT 960 also identified comparable proportion 15.9% (11/69) as drug resistant with 8 and 3 samples as from the new and retreatment ones, respectively. More than half of the sub-culture positive samples by MIGIT 56.5% (39/69) were from Dessie referral hospital and all isolates from BMH were detected as drug resistant by either MGIT, LPA or both.

Drug resistance was detected in 8 common isolates of MGIT and LPA test. In addition, the LPA identified 2 isolates as susceptible which were resistant by MGIT and also no TUB band was observed in LPA strip for one isolate. Six of the resistant isolates detected by LPA were not tested for their sensitivity by MGIT due to their negative sub-culture result and 2 of the isolates resistant by LPA were susceptible to MGIT.

**Fig. 2:**
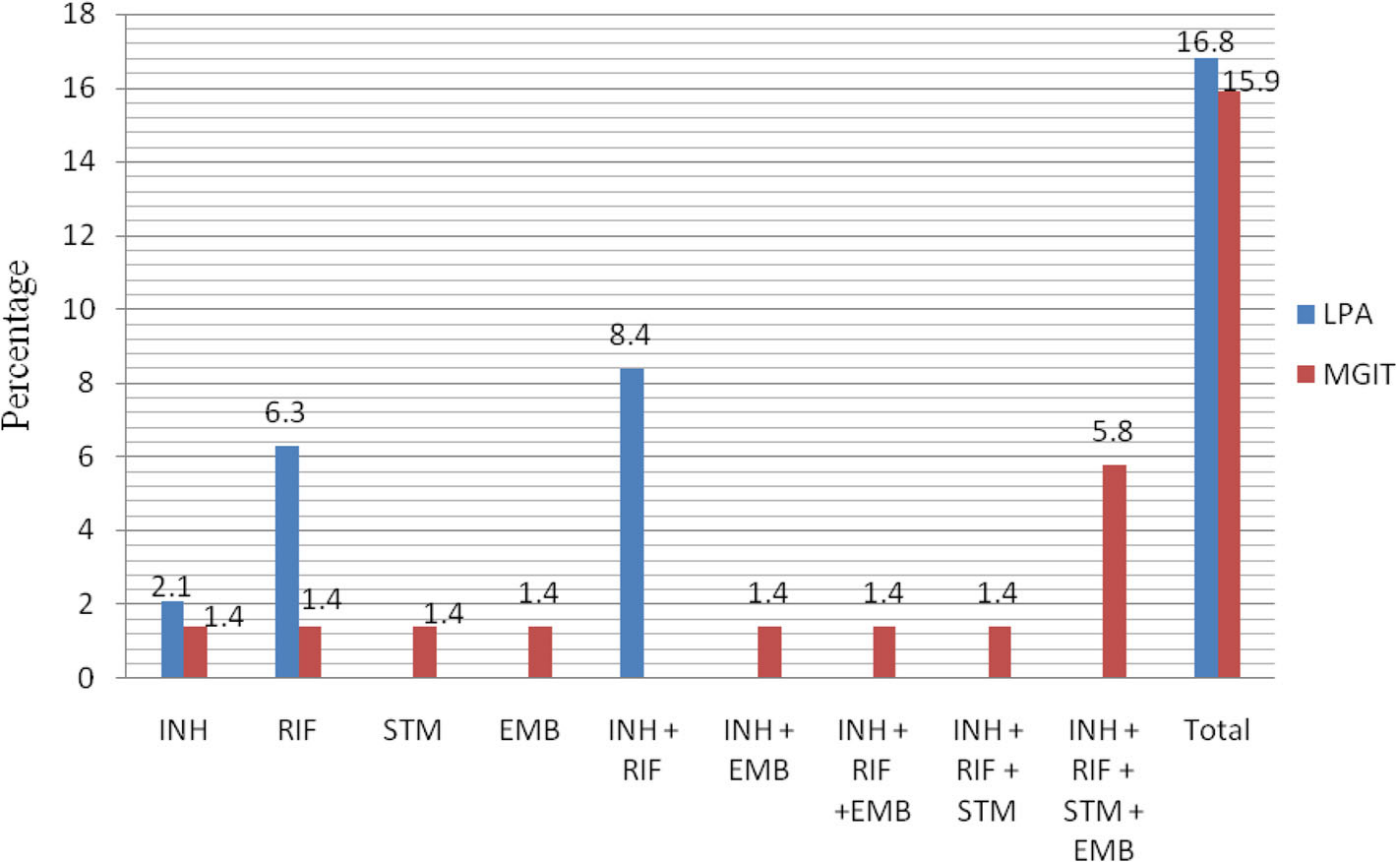
Drug resistance pattern by genotypic line probe assay (LPA) and phenotypic *Mycobacterium* growth indicator tube (MGIT) from northeastern Ethiopia, April 2015 to January 2017. INH Isoniazid, RIF Rifampicin, STM Streptomycin, EMB Ethambutol, LPA line probe assay, MGIT *Mycobacterium* growth indicator tubes

Greater proportion 8.4% (8/95) of the LPA tested isolates were multidrug resistance than mono-resistant. Of these, 5.3% (5/95) was among new patients and 3.2% (3/95) of the MDR as among retreatments. Rifampicin and isoniazid mono-resistance accounted for 6.3% (6/95) and 2.1% (2/95), respectively. The LPA test detects 57.1% (8/14) of rifampicin resistant isolates had multidrug resistant TB. On the other hand, all of the sub-culture positive samples by BACTEC MGIT 960 underwent 276 tests and 15.9% (11/69) of the samples were detected as resistant to any of the four anti-TB drugs. More resistance was detected 5.8% (4/69) to the four first-line antimicrobial drugs (INH + RIF + STM + EMB) by the MGIT test than their mono-resistance (INH =1.4%, RIF =1.4%, STM = 1.4%, EMB = 1.4%) (Fig. 2). The frequency of resistance to isoniazidniazd, rifampicin, streptomycin and ethambutol was in a comparable proportion 11.6% (8/69), 10.1% (7/69), 10.1% (7/69) and 8.7% (6/69); in that order. Greater proportion of resistance was detected in females 22.6% (7/31) than males 14.1% (9/64) by LPA test and also comparable proportion of drug resistance was detected in females 20% (4/20) than males 14.3% (7/49) by MGIT.

As compared to BACTEC MGIT 960, the sensitivity and specificity of the GenoType MTBDR*plus* assay for the detection of RIF-resistant *M. tb* isolates was 100 and 98.3%, respectively. Similarly, the sensitivity and specificity for detection of INH-resistance was 75 and 98.2%, and for MDR resistance 83.3 and 100%, respectively. Kappa value showed that there is almost a perfect agreement between the two methods in detecting RIF and MDR where as there is a substantial agreement for the detection of isoniazid (Table 2).

**Table 2:** Performance of GenoTypic MTBDR*plus* assay for detection of RIF, INH and MDR resistance in comparison to phenotypic BACTEC MGIT 960 System

Drugs	MTBDR***plus***	BACTEC MGIT 960		Sensitivity(%)	Specificity(%)	PPV(%)	NPV(%)	Kappa
		Resistant	Sensitive					
**RIF**	Resistant	7	1	100	98.3	87.5	100	0.925 (*p* < 0.001)
	Sensitive	0	57					
**INH**	Resistant	6	1	75	98.2	85.7	96.6	0.774 (*p* < 0.001)
	Sensitive	2	56					
**MDR**	Resistant	5	0	83.3	100	100	98.3	0.901 (*p* < 0.001)
	Sensitive	1	59					

In comparison to MGIT, the drug sensitivity of LPA at *katG* gene was found as 75% detection and that of *inhA* promoter region was 12.5%. The sensitivity of LPA to both *katG* and *inhA* was also 12.5%. This finding also revealed that the *katG*, *inhA*, both *katG* and *inhA* results of LPA test had almost perfect agreement, of susceptibility to the MGIT test (Table 3).

**Table 3:** Detection of isoniazid (katG, inhA and both katG and inhA) resistance by LPA with its resistance by MGIT testing method

LPA		MGIT isoniazid DST		Sensitivity	Specificity	Predictive value		Kappa
		Resistant = 8	Sensitive = 57			Positive	Negative	
*katG*	Resistant	6	0	75	100	100	96.6	0.840 (*P* < 0.001)
	Sensitive	2	57					
*inhA*	Resistant	1	1	12.5	98.2	50	88.9	0.159 (*P* = 0.099)
	Sensitive	7	56					
*KatG* and *inhA*	Resistant	1	0	12.5	100	100	89.1	0.2 (*P* = 0.007)
	Sensitive	7	57					

### Resistance and mutation patterns of rifampicin and isoniazid using the GenoType MTBDR*plus* assay

For the line probe assay*,* single probe resistance was detected in 25.0% (4/16) of the isolates and two or more probe resistance was detected in the remaining drug resistant isolates. In addition, single probe mutation was detected in 12.5% (2/16) of the drug resistant isolates. In 10 of the RIF resistant isolates, the WT8 was missed with an additional miss of WT3, WT4 and WT6 probe in the *rpoB* gene of the three isolates and no mutation was detected. There were also an omission of the *rpoB*WT3 and WT7 probe for 3 RIF resistant isolates with unknown amino acid change for all the 13 RIF resistant isolates in the *rpoB* gene region. Concerning isonizid resistance, the resistance was further identified using *katG* and *inhA* gene region. Of the 12 INH-resistant isolates, *katG* resistance was detected in 66.7% (8/12) of the isolates by omitting WT probe at codon 315 and mutations were detected in two of the isolates at Codon S315T1. Mutation was identified in five isolates of the *inhA* gene region at WT1 (− 15/− 16) and an additional mutation to one isolate at WT2 (− 8). Addition of specific *inhA* mutations were not observed in place of the omitted probes at the *inhA* promoter region of the gene.

### Genetic diversity for the drug resistant *Mycobacterium tuberculosis*

Of the major lineages identified using spoligotyping 76.5% (13/17) were Euro-American followed by Indo-Oceanic 17.6% (3/17) lineages. Most of the drug resistant isolates 76.5% (13/17) had no SIT number indicating that they are orphans. Family 33 is also the predominant family identified in this study (Table 4).

**Table 4:** Spoligotyping result of drug resistant isolates from northeastern Ethiopia

Resistant Isolates	Family	Major Lineage by CBN	sub-lineage /clade	SIT
BHDL007	*M. tuberculosis* Haarlem3	Euro-American	H3	1802
BHDL020	*M. tuberculosis* T1	Euro-American	Manu3	
BHDL030	Family 33	Euro-American	Manu2	
BMH08	*M. tuberculosis* H37Rv	Euro-American	T	
BMH09	*M. tuberculosis* T3	Euro-American	T3-ETH	
BMH13	*M. tuberculosis* CAS	East-African-Indian	CAS1-Kili	
BMH23	*Family 33*	Euro-American	Manu2	
BTHC09	*M. tuberculosis* T3	Euro-American	T1-RUS2	
CRHC02	*Family33*	Indo-Oceanic	Manu1	
DHC21	Family 33	Euro-American	T4	
DRH021	*M. tuberculosis* T1	Euro-American	T	612
DRH030	Family33	Euro-American	Manu2	1088
DRH032	*M. tuberculosis* EAI4	Indo-Oceanic	CAS1-Kili	
DRH038	*M. tuberculosis* Haarlem3	Euro-American	X1	
DRH046	*M. tuberculosis* T1	Euro-American	Manu3	
DRH122	*M. tuberculosis* Haarlem1	Euro-American	H1	47
DRH123	*M. tuberculosis* Haarlem1	Indo-Oceanic	H1	

The association between any drug resistance and TB type showed a varied proportion but with no statistically significant difference among pulmonary and extra-pulmonary TB cases (χ2: 0.233; *p* = 0.629). A patient with previous TB treatment and drug-resistance linked to new cases infected with the same strain (Table 5).

**Table 5:** Association between any drug sensitivity patterns and TB type, major lineage, sub-lineage and dominant strains

Variable	Any drug resistance				
	Sensitive (%)	Resistant (%)	Total (%)	χ2 (df)	*P*- value
**TB type**					
Extra-pulmonary	10 (76.9%)	3 (23.1%)	13 (14%)	0.233 (1)	0.629
pulmonary	66 (82.5%)	14 (17.5%)	80 (86%)		
**Major lineage by CBN**					
EA	47 (79.7%)	12 (20.3%)	59 (63.4%)	0.757 (2)	0.757
IO	15 (83.3%)	3 (16.7%)	17 (19.4%)		
EAI	14 (87.5%)	2 (12.5%)	16 (17.2%)		
**Sub-lineage/Clade**					
AFRI	4 (100%)	0	4 (4.3%)		
CAS	3 (100%)	0	3 (3.4%)		
CAS1-Delhi	12 (100%)	0	12 (12.8%)		
CAS1-Kili	2 (40.0%)	3 (60.0%)	5 (5.3%)		
EAI1-SOM	1 (100%)	0	1 (1.1%)		
H	3 (100%)	0	3 (3.4%)		
H1	0	2 (100%)	2 (2.1%)		
H3	2 (66.7%)	1 (33.3%)	3 (3.4%)		
H3-Ural-1	1 (100%)	0	1 (1.1%)		
H37Rv	1 (100%)	0	1 (1.1%)		
H4-Ural-2	3 (100%)	0	3 (3.4%)		
Manu_ancestor	0	1 (100%)	1 (1.1%)	34.861(22)	0.040
Manu1	3 (75%)	1 (25%)	4 (4.3%)		
Manu2	6 (75%)	2 (25%)	8 (8.5%)		
Manu3	7 (70%)	3 (30%)	10 (10.6%)		
PINI	1 (100%)	0	1 (1.1%)		
T	17 (94.4%)	1 (5.6%)	18 (19.1%)		
T-Tuscany	1 (100%)	0	1 (1.1%)		
T1-RUS2	0	1 (100%)	1 (1.1%)		
T3	1 (100%)	0	1 (1.1%)		
T3-ETH	6 (85.7%)	1 (14.3%)	7 (7.4%)		
T4	2 (66.7%)	1 (33.3%)	3 (3.4%)		
Turkey	1 (100%)	0	1 (1.1%)		
**Dominant strains**					
Orphan	66 (86.8%)	13 (76.5%)	79 (84.9%)		
SIT1166	1 (1.3%)	0 (0.0%)	1 (1.1%)		
SIT1251	1 (1.3%)	0 (0.0%)	1 (1.1%)		
SIT1378	1 (1.3%)	1 (5.9%)	2 (2.2%)		
SIT1475	1 (1.3%)	0 (0.0%)	1 (1.1%)	16.948(10)	0.076
SIT149	3 (3.9%)	0 (0.0%)	3 (3.2%)		
SIT1547	1 (1.3%)	0 (0.0%)	1 (1.1%)		
SIT1802	0 (0.0%)	1 (5.9%)	1 (1.1%)		
SIT47	0 (0.0%)	1 (5.9%)	1 (1.1%)		
SIT53	2 (2.6%)	0 (0.0%)	2 (2.2%)		
SIT612	0 (0.0%)	1 (5.9%)	1 (1.1%)		
**Clustering**					
Yes	10 (62.5%)	6 (37.5%)	16 (17.2)	4.779 (1)	0.029
No	66 (85.7%)	11 (14.3%)	77 (82.8)		

## Discussion

### GenoTypic and phenotypic drug sensitivity test

The microbiological drug sensitivity test using GeneXpert is crucial to treat the RIF resistant (a surrogate marker for MDR-TB) patients on the spot than the other time taking testing methods. The additional detection of drug resistant isolate using MTBDR*plus* assay and BACTEC MGIT 960 test was due to single detection of resistance by the Xpert (only RIF resistant cases). The GenoTypic LPA can detect an additional INH resistance where as the phenotypic MGIT can detect even a more additional drugs such as STM and EMB to RIF and INH. This could enhance the chance of detecting more resistant isolates. The lower proportion of MGIT sub-culture positivity detection 61.6% (69/112) for DST test from LJ-culture positive *Mycobacterium* sample than the heat killed LPA isolates 84.8% (95/112) might be due to the existence of bacterial DNA in the later one. In the case of freezed live *Mycobacterium*, its survival could be interrupted through time until it was sub-cultured using the MGIT instrument. The higher sensitivity result 94.2% of LPA and its lower specificity value 30.2% is comparable with similar settings done in Kenya where its sensitivity and specificity was 99.2 and 26.9%, respectively [17]. This showed that LPA has a good performance in detecting the true positivity of *Mycobacterium* and in a fair agreement (Kappa = 0.276; *P* < 0.001) with the BACTEC MGIT 960 performance.

The overall genotypic drug sensitivity test in this study detected 16.8% (16/95) as drug resistant to either of RIF and/or INH. This is higher than 7.7% (10/130) of similar studies done by using LPA in Arsi South central Ethiopia [18], 8.7% (14/161) and 13.3% (37/279) reported from central Ethiopia [19]. In contrast, our finding is in less proportion than a study report from northwest Ethiopia [20] and southwest Ethiopia [21] having a rate of 18.0% (20/111) and 39.3% (44/112), respectively. Studies from Chad [22] and Punjab state of India [23] also reported greater proportion of resistance as 23.4% (73/312) and 58.4% (163/279), respectively. The possible explanation for such variations of drug resistant proportion could be due to difference in sample size, study sites and subjects where the samples were collected.

The rate of MDR in this study was 8.4% (8/95) by genotypic MTBDR*plus* assay which is greater than 1.1% (3/279) [19], 1.8% (2/111) [20], 3.1% (5/161) [24] and 6.7% (11/165 [25] similar studies. On the contrary, our MDR finding is less than, 17.5% (33/189) [26], 25.8% (72/279) [23] and 27.7% (31/112) [21]. Regarding the detection of RIF 6.3% (6/95) and INH 2.1% (2/95) mono-resistance, the current prevalence in line with a number of studies [27–29] that rifampicin mono-resistant is greater than isoniazid mono-resistant with varying degrees of proportion. Such RIF resistance is an important implication for higher risk of multi-drug resistance as it is a surrogate marker of MDR [26]. Nevertheless, there are also studies [19, 20] that show isoniazid mono-resistance as the leading mono-resistant than RIF.

The 15.9% (11/69) drug resistant detection rate using BACTEC MGIT 960 in this study was at greater proportion than similar studies [30, 31]. On the contrary, less proportion of drug resistances were reported in this study than other research reports by using the same detection system [32, 33] which might be due to different study areas and sample size. Most of the isolates detected as resistant by LPA were also resistant by MGIT. In 2 of the samples which were detected as resistant by MGIT but not by LPA could be due to its extra drug sensitivity detection test. All the MDR resistant samples by MGIT in this study were not restricted to isoniazid and rifampicin, but had an additional resistance to EMB, STM or both. This implied sensitivity detection by MGIT posses an extra benefit to detect other first line TB drugs.

The high sensitivity of the MTBDR*plus* assay (100%) in detecting RIF resistance in this study is the same with similar study settings in Southern [34] and northwestern [35] Ethiopia, but with little variation to the isolates from central Ethiopia [19]. The specificity of the assay (98.3%) in detecting RIF resistance is comparable to 99.2% [34], 99.8% [19] and 100% [35] in detecting RIF resistant isolates. There is a great variation of MTBDR*plus* assay sensitivity to INH in our study 75% and a study from the Southern Ethiopia 33.3% [34]. The sensitivity of the assay to INH was 82.7 in a study of isolates from central [19] and 91.7% in isolates collected from northwestern part of the country [35]. The susceptibility of MTBDR*plus* to INH in this finding 98.2% corresponds to similar reports (97.2–100%) in the country. Although the assay’s sensitivity result to detect MDR have some discrepancy, the susceptibility result accounts 100% which *coincides exactly* with other studies reported from the country [19, 34, 35].

The study also found that there was an excellent agreement between BD BACTEC MGIT 960 and MTBDR*plus* assay in detecting RIF and MDR with a Kappa value of 0.925 and 0.901, respectively. However, it was found as a good agreement (Kappa value, 0.774) between the two testing methods in detecting INH. Similarly, it was also reported as an excellent agreement in detecting MDR (Kappa value, 1) but with good (0.663) and moderate (0.494) agreement in detecting RIF and INH resistance, respectively [34]. Such difference between the two findings might be due to the difference in number of drug resistant isolates. Further, the agreement between Culture MGIT and LPA in detecting *katG*, *inhA* and both (*katG* and *inhA*) resistance was almost perfect and agrees with a study from Kenya [17].

### Frequency of gene mutations associated with rifampicin and isoniazid resistant isolates by GenoType MTBDR*plus* assay

The wild-type *rpoB* probe hybridization band pattern showed an omission of the bands at WT3, WT4, WT6, WT7 and WT8 probes. Of these, the miss of WT8 probe predominates and accounted for 71.4% (10/14) among the RIF resistant isolates in a small region of amino acids located between position numbers 530–533 of the *rpoB* gene. Omission of all the *rpoB* gene probes was without any gain of probes in the MUT region and depicted as “unknown” mutation. Such lack of binding of a WT probe without simultaneous binding of a mutant probe is likely caused by the presence of a resistant mutation. Similarly, missing of probes without any gain of the corresponding MUT was reported from northwestern Ethiopia [20]. A report from central Ethiopia also in line with our finding that no gain of any mutant band was identified for the non-hybridized WT8 probe [19]. Similar findings were also reported for the absence of MUT band from other countries [36, 37]. Despite such reports, there were research findings where gain of MUT probes was identified in a part of the *rpoB* gene [21].

Greater frequency of resistance 8/16 (50%) to INH occurred due to mutation of the *katG* gene, whereas lower frequency of resistance 5/16 (31.3%) was caused by the mutations in the promoter region of the *inhA* gene. Greater frequencies of the *katG* gene WT omission at codon 315 was also reported from other studies in Ethiopia that agrees with this finding [19–21]. A miss of WT probe at Codon 315 in the *katG* gene without the presence of specific MUT band accounted 75% (6/8) in this study. The remaining 25% of the strains had mutations in the *katG* gene at codon 315 with amino acid change of S315T1 (AGC → ACC). The *inhA* promoter region showed a miss of − 15/− 16 codon that was detected in 5/16 (31.3%); and 1/16 (6.3%) at − 8 gene region without conferring any addition of specific mutational band in both cases. This mutation also agrees with the finding from central Ethiopia and also considered as ‘unknown’ mutation [19].

### Genetic diversity of the drug resistant *Mycobacterium tuberculosis*

The higher proportion of Euro-American lineages among drug resistance strains could be due to its higher prevalence 63.4% (59/93) under the interpretable spoligotyping results of the LPA done isolates in this study. This in line with findings that reported greater proportion of the same most frequently reported major lineage from other parts of Ethiopia [20, 38]. The clustered strains of the study also showed higher frequency and a statistically significant association with any anti-TB drug resistance than the unique ones revealing an implication for the highest risk of drug resistance among recent TB transmission.

## Conclusions

Resistance of *M. tb* to common drugs showed agreeable patterns in both conventional phenotypic BD BACTEC MGIT 960 and the GenoTypic MTBDR*plus* assay test. Both testing methods highlighted greater proportion of drug resistant isolates among newly treated patients than retreatment ones. Rifampicin mono-resistance was accounted for greater proportion of resistance than INH mono-resistance in the study and the majority of the RIF resistant isolates were also found as multi-drug resistant. Euro-American major lineages as the predominant lineage with most of the drug resistant isolates as orphans. Thus, further consideration should be given toward drug sensitivity testing methods in a large number of LJ-positive samples and to identify the circulating strains for better prevention and control programs which might realizes the targeted “End TB strategy”.

### Limitations

In this study, the less number of culture positivity from large smear positive samples due to various reasons and lack of second line drug sensitivity test was considered as its limitation.

## Data Availability

All the data sets on which our conclusions relayed on were presented in the main section of this manuscript.

## Abbreviations

AC: Amplification control
ALIPB: Aklilu Lemma Institute of Pathobiology
BHDL: Biqat higher diagnostic laboratory
BMH: Boru Meda hospital
BTHC: Bati Town health centre
CBN: Conformal Bayesian network
CRHC: Chefa Robit health center
DHC: Dessie health center
DNA: Deoxyribonucleic acid
DOT: Directly observed therapy
DRH: Dessie referral hospital
DST: Drug sensitivity test
EA: Euro-American
EAI: East-African-Indian
EMB(E): Ethambutol
EPTB: Extra-pulmonary tuberculosis
FNA: Fine needle aspirate
GC: Growth control
GU: Growth unit
HIV: Human Immunodeficiency Virus
INH(I): Isoniazid
IO: Indo-Oceanic
IQR: inter-quartile range
KBBN: Knowledge based Bayesian network.
LJ: Löwenstein Jensen
LPA: Line probe assay
*M. tb*: *Mycobacterium tuberculosis*
MDR: Multi-drug resistance
MGIT: *Mycobacterium* growth indicator tubes
MUT: Mutant
NPV: Negative predictive value
PCR: Polymeraze chain reaction
PPV: Positive predictive value
PZA(Z): Pyrazinamide
RIF(R): Rifampicin
RNA: Ribonucleic acid
RR: Rifampisin resistance
SD: Standard deviation
SIT: Spoligotype International Type
Spoligotyping: Spacer Oligonucleotidetyping
SPSS: Statistical Package for the Social Sciences
STM(S): Streptomycin
TB: Tuberculosis
WHO: World Health Organization
WT: Wild type
XDR: Extensively drug-resistant
